# Ozone’s Impact on Public Health: Contributions from Indoor Exposures to Ozone and Products of Ozone-Initiated Chemistry

**DOI:** 10.1289/ehp.9256

**Published:** 2006-06-08

**Authors:** Charles J. Weschler

**Affiliations:** 1 Environmental and Occupational Health Sciences Institute, University of Medicine and Dentistry of New Jersey–Robert Wood Johnson Medical School and Rutgers University, Piscataway, New Jersey, USA; 2 International Centre for Indoor Environment and Energy, Technical University of Denmark, Lyngby, Denmark

**Keywords:** air exchange rates, aldehydes, indoor chemistry, inhalation intake, morbidity, mortality, secondary organic aerosols, surface chemistry, ultrafine particles

## Abstract

**Objective:**

The associations between ozone concentrations measured outdoors and both morbidity and mortality may be partially due to indoor exposures to ozone and ozone-initiated oxidation products. In this article I examine the contributions of such indoor exposures to overall ozone-related health effects by extensive review of the literature as well as further analyses of published data.

**Findings:**

Daily inhalation intakes of indoor ozone (micrograms per day) are estimated to be between 25 and 60% of total daily ozone intake. This is especially noteworthy in light of recent work indicating little, if any, threshold for ozone’s impact on mortality. Additionally, the present study estimates that average daily indoor intakes of ozone oxidation products are roughly one-third to twice the indoor inhalation intake of ozone alone. Some of these oxidation products are known or suspected to adversely affect human health (e.g., formaldehyde, acrolein, hydroperoxides, fine and ultrafine particles). Indirect evidence supports connections between morbidity/mortality and exposures to indoor ozone and its oxidation products. For example, cities with stronger associations between outdoor ozone and mortality tend to have residences that are older and less likely to have central air conditioning, which implies greater transport of ozone from outdoors to indoors.

**Conclusions:**

Indoor exposures to ozone and its oxidation products can be reduced by filtering ozone from ventilation air and limiting the indoor use of products and materials whose emissions react with ozone. Such steps might be especially valuable in schools, hospitals, and childcare centers in regions that routinely experience elevated outdoor ozone concentrations.

Many studies have reported associations between outdoor ozone concentrations and morbidity and mortality. Hubbell et al. (2005) systematically summarized this literature, including associations between ozone and respiratory-related hospital admissions, lost school days, restricted activity days, asthma-related emergency department visits, and premature mortality. Additionally, ozone has been associated with respiratory symptoms and the use of asthma medication for asthmatic school children using maintenance medication (Gent et al. 2003), and long-term exposure to ozone has been tentatively associated with the development of asthma in adult males (McDonnell et al. 1999). Since the submission of Hubbell et al. (2005), three independent meta-analyses have been published, indicating an increase of 0.87% in mortality per 10-ppb increase in daily ozone (Bell et al. 2005), an increase of 0.39% in mortality per 10-ppb increase in 1-hr daily maximum ozone (Ito et al. 2005), and an increase of 0.41% in mortality per 10-ppb increase in 1-hr daily maximum ozone (Levy et al. 2005); in most of the studies included in the meta analyses, same-day effects were larger than lagged effects. A study of 23 European cities found an increase of 0.66% in mortality per 10 ppb increase in 1-hr maximum ozone during the summer (Gryparis et al. 2004); a study in Genoa, Italy, found an increase of 4.0% in mortality per 25-ppb increase in ozone (Parodi et al. 2005); and a study in Shanghai found an increase of 0.45% in mortality per 5-ppb increase in 2-day average ozone (Zhang et al. 2006). Significantly, even when Bell et al. (2006) used data that included only days with average ozone levels lower than 15 ppb, outdoor ozone was significantly associated with premature mortality. For a more extended review of these and other studies, see the U.S. Environmental Protection Agency ozone criteria document (U.S. EPA 2006).

An increase in the concentration of outdoor ozone concomitantly produces an increase in the indoor concentrations of ozone and its reaction products (Weschler 2000). Thus, some of the associations between outdoor ozone and both morbidity and mortality are likely due to outdoor ozone transported into various indoor environments (e.g., residences, workplaces, schools, hospitals, motor vehicles) where subsequent exposures occur. Although indoor ozone concentrations tend to be smaller than corresponding outdoor concentrations, this is somewhat counterbalanced by the much larger fraction of time that most people spend indoors. Moreover, excepting nitrogen dioxide, total concentrations of ozone reaction products are anticipated to be larger indoors than outdoors (see “Products of ozone-initiated indoor chemistry”).

My aim in this article is to present evidence supporting the hypothesis that indoor exposures to ozone and its oxidation products contribute to ozone’s overall impact on public health. Apportioning ozone’s health impact among indoor and outdoor ozone, as well as indoor and outdoor oxidation products, is more than an academic exercise. If indoor ozone and the products of its chemistry are adversely affecting the public’s health, relatively simple strategies can mitigate these effects.

## Indoor Ozone: Exposures and Intakes

### Indoor ozone concentrations

Although there are indoor sources of ozone, in most buildings indoor ozone has been transported from outdoors (Weschler 2000). Indoor ozone concentrations track outdoor concentrations with a slight time lag that depends on the air exchange rate. Ozone is removed by indoor surfaces as well as by gas-phase reactions, and hence, indoor concentrations tend to be smaller than co-occurring outdoor levels. Models have been presented that relate indoor ozone concentrations to those outdoors (Nazaroff and Cass 1986; Sabersky et al. 1973; Shair and Heitner 1974). In the absence of indoor sources, the ratio of indoor to outdoor ozone concentrations (I:O) can be estimated using a relatively simple expression (Weschler et al. 1989):


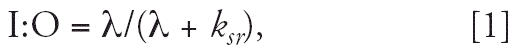


where λ is the air exchange rate and *k_sr_* is the first-order rate constant for surface removal (both in units of reciprocal time). This equation assumes that the penetration coefficient for ozone is unity, an assumption that remains largely untested, and ignores gas-phase reactions, which tend to be smaller sinks than surface reactions. Numerous investigators have measured surface removal rate constants for ozone, as summarized in several reviews (Grontoft and Raychaudhuri 2004; Nazaroff et al. 1993; Weschler 2000). For example, Lee et al. (1999) found a mean value of 2.8 ± 1.3 hr^−1^ in 43 Southern California homes. A review of air exchange rates in residences and nonresidences has recently appeared in a draft U.S. EPA report (U.S. EPA 2005). Air exchange rates vary with the region and the season. In general, mean residential values are between 0.6 and 1.7 hr^−1^, and mean nonresidential values are between 1.5 and 2.0 hr^−1^. If a value of 3 hr^−1^ is used for *k_sr_*, Equation 1 predicts an I:O of 0.10 at an air exchange rate of 0.33 hr^−1^, an I:O of 0.33 at 1.5 hr^−1^ and an I:O of 0.50 at 3 hr^−1^. These calculated estimates are consistent with measured values, for example, a mean I:O of 0.37 ± 0.25 at 126 Southern California homes (Avol et al. 1998), and a mean I:O of 0.20 ± 0.18 at 145 homes in Mexico City and I:O values between 0.3 and 0.4 at three corresponding schools during class hours (Romieu et al. 1998); see table 2 of Weschler (2000) for a more extensive summary of measured I:Os for ozone.

Residences with central air conditioning (AC) have I:Os that are typically < 0.10 (Lee et al. 1999, 2004; Stock et al. 1985). This reflects the fact that outdoor air is not deliberately introduced in air-conditioned residences and that the outdoor air exchange due to leakage is typically quite small. Additionally, filters used in air conditioners remove some of the ozone from the air that passes through them (Bekö et al. 2006; Hyttinen et al. 2003, 2006).

### Indoor versus outdoor exposures and intakes

**Figure 1:**
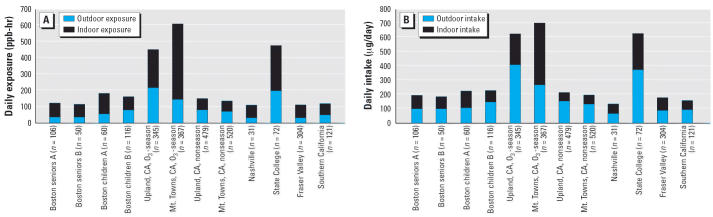
(*A*) Calculated indoor and outdoor ozone exposures. (*B*) Calculated indoor and outdoor ozone intakes (see text for details). Data from Boston, Massachusetts: Sarnat et al. (2005); Upland and Mt. Towns, California: Geyh et al. (2000); Nashville, Tennessee: Lee et al. (2004); State College, Pennsylvania: Liu et al. (1993); Fraser Valley, British Columbia, Canada: Brauer and Brook (1995); Southern California: Linn et al. (1996).

“Exposure” to an air contaminant in a given microenvironment has been defined as the concentration of a pollutant in that microenvironment times the amount of time an individual spends there (National Academy of Sciences 1991). Early estimates (Weschler et al. 1989) suggested that ozone exposures occurring indoors are comparable to those occurring outdoors, especially for individuals such as the very young, very old, or chronically ill who spend very little time outdoors. Based on indoor measurements made in six New Jersey homes, Zhang and Lioy (1994) estimated that indoor exposures accounted for more than half the total exposure for the occupants of these homes. In the intervening years, several studies have used passive ozone monitors to measure personal ozone concentrations—ozone concentrations experienced by an individual throughout a 24-hr period—and have made comparisons between these measurements and ozone concentrations measured at outdoor fixed site monitors (Brauer and Brook 1995; Geyh et al. 2000; Lee et al. 2004; Linn et al. 1996; Liu et al. 1993; Sarnat et al. 2005). To a first approximation, the average personal ozone concentration measured in these studies is given by


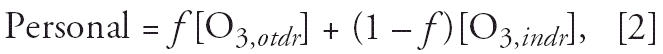


where *f* is the fraction of time outdoors, [O_3,_*_otdr_*] is the average outdoor ozone concentration while outdoors, 1 − *f* is the fraction of time indoors, and [O_3,_*_indr_*] is the average indoor ozone concentration while indoors. Figure 1A shows average daily outdoor and indoor ozone exposures, in units of parts per billion per hour, estimated from the studies cited above (each includes sufficient information on the parameters in Equation 2 to enable calculations of these estimates; time in transit has been categorized as time indoors). For the studies shown in Figure 1A, indoor exposures are 43–76% of total daily ozone exposure, with an average of just below 60%.

Figure 1A does not account for the fact that breathing rates for both children and adults vary with activity levels and, on average, tend to be higher outdoors. An individual’s daily ozone inhalation intake (micrograms per day) is estimated by


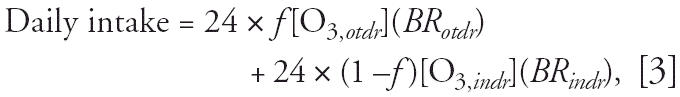


where *BR_otdr_* and *BR_indr_* are the average breathing rate while outdoors and indoors, respectively. Figure 1B shows estimates of daily outdoor and indoor ozone intakes (micrograms per day) calculated using Equation 3. In calculating these intakes, breathing rates corresponding to light exercise (0.95 m^3^/hr for children, 1.39 m^3^/hr for adults) were used for *BR_otdr_*, and breathing rates corresponding to sedentary activity (0.47 m^3^/hr for children, 0.54 m^3^/hr for adults) were used for *BR_indr_* (U.S. EPA 1997). Total breathing rates were used rather than fractional rates corresponding only to alveolar ventilation, because most of the inhaled ozone reacts with ascorbic acid, uric acid, glutathione, and unsaturated fatty acids present in the epithelial lining fluid in the conducting airways (Postlethwait and Ultman 2001; Rigas et al. 2000). Although outdoor ozone intakes in Figure 1B tend to be larger, indoor ozone intakes account for between 27 and 60% of total daily ozone intake and average just over 40%. Given that even low levels of ozone have been associated with increased risk of premature mortality (Bell et al. 2006), indoor intakes should not be ignored.

## Products of Ozone-Initiated Indoor Chemistry: Exposures and Intakes

### Indoor sources of ozone-reactive chemicals

Indoor exposure to ozone is accompanied by exposure to the products of ozone-initiated indoor chemistry (Weschler 2004). In general, these products are a consequence of ozone reacting with many commonly found organic chemicals that contain unsaturated carbon–carbon bonds (e.g., isoprene, styrene, terpenes, sesquiterpenes, squalene, and unsaturated fatty acids and their esters) because such compounds react with ozone much faster than do saturated organic compounds. Table 1 is a summary common indoor sources of ozone-reactive chemicals, including the occupants themselves, soft woods, carpets, linoleum, certain paints, polishes, cleaning products and air fresheners, soiled fabrics, and soiled ventilation filters. These ubiquitous sources result in substantial quantities of indoor chemicals that can react with ozone whenever outdoor concentrations are elevated.

### Products of ozone-initiated indoor chemistry

There are several reasons why products of ozone-initiated chemistry tend to have higher concentrations indoors than outdoors. First, there are more ozone-reactive chemicals indoors than outdoors because of the presence of consumer products, architectural coatings, furnishings, and building materials; indeed, some sources occur almost exclusively indoors (e.g., carpets, linoleum, air fresheners). Second, the concentrations of ozone-reactive compounds tend to be higher indoors than outdoors (Brown et al. 1994; Hodgson and Levin 2003; Wolkoff 1995), reflecting more sources and larger emission rates per volumetric flow rate. Third, surface-to-volume ratios (S:V) are roughly two orders of magnitude larger indoors than outdoors based on characteristic mixing heights outdoors (Nazaroff et al. 2003). This is partially counterbalanced by higher deposition velocities, *v_d_*, outdoors (Finlayson-Pitts and Pitts 2000) compared with indoors (Weschler 2000). On average, the first-order rate constant that describes surface removal (the product of S:V and *v_d_*) is about 30 times larger indoors than outdoors. Indoor surface reactions are major sources of oxidation products (Destaillats et al. 2006b; Fick et al. 2004; Morrison and Nazaroff 2002; Weschler et al. 1992b; Wisthaler et al. 2005). Additionally, unlike gas-phase reactions (Weschler and Shields 2000), surface chemistry can include reactions whose rates are slower than air exchange rates.

Table 1 is a summary of the major stable reaction products anticipated from indoor ozone chemistry. Evidence from field studies suggests that indoor concentrations of some of these stable products (e.g., organic acids and carbonyls) correlate with ozone concentrations (Bako-Biro et al. 2005; Reiss et al. 1995b; Zhang et al. 1994). In addition to stable products, ozone chemistry produces relatively short-lived products. Examples include primary and secondary ozonides, peroxyhemiacetals, α-hydroxy ketones, α-hydroxy hydroperoxides, and peroxyacyl nitrates (Atkinson and Arey 2003; Docherty et al. 2005; Finlayson-Pitts and Pitts 2000; Norgaard et al. 2006; Ziemann 2003). Although short-lived, many of these products exist long enough to be inhaled and transported into the respiratory tract. Indoor ozone also reacts with alkenes to produce hydroxyl radicals (Destaillats et al. 2006a; Fan et al. 2003; Sarwar et al. 2002; Weschler and Shields, 1996, 1997) and with nitrogen dioxide to produce nitrate radicals (Weschler et al. 1992a). These are highly reactive oxidants in their own right. Indeed, at typically anticipated indoor concentrations, ozone-derived nitrate radicals react much faster with alkenes and polycyclic aromatic hydrocarbons (PAHs) than with ozone alone [see table 8 of Nazaroff and Weschler (2004)].

Secondary organic aerosols (SOAs), consisting of primarily fine and ultrafine particles, are an important subgroup of stable products resulting from ozone-initiated chemistry. They are formed from low-vapor pressure–oxidation products that partition between the gas phase and the surface of preexisting particles or nucleate to form new aerosols. The reaction of ozone with various terpenoids in indoor settings has been shown to contribute tens of micrograms per cubic meter to the indoor concentration of submicrometer particles under appropriate conditions (Destaillats et al. 2006a; Fan et al. 2003, 2005; Long et al. 2000; Rohr et al. 2003b; Sarwar et al. 2003, 2004; Wainman et al. 2000; Weschler and Shields 1999).

### Studies indicating indoor exposure to SOAs from ozone-initiated chemistry

Particulate organic carbon was analyzed in samples of indoor and outdoor fine particles collected at 173 homes in Houston, Texas; Los Angeles, California; and Elizabeth, New Jersey. At least 40%, but more likely 70–75%, of the particulate organic carbon associated with indoor particles was generated indoors (Polidori et al. 2006). The authors speculated that a portion of this may have been contributions from SOAs generated by indoor ozone chemistry. Table 2 presents data from Sarnat et al. (2005) that support the concept that SOAs generated indoors can make meaningful contributions to personal exposures to particles < 2.5 μm in diameter (PM_2.5_). The table shows personal (P) and corresponding outdoor (O) concentrations of PM_2.5_ and fine-mode sulfate (SO_4_^2−^) for senior citizens living in Boston, Massachusetts, during five monitoring periods. The fine-mode sulfate concentrations are derived from analyses of the PM_2.5_ filters and serve as markers for personal exposure to PM_2.5_ of outdoor origin because fine-mode sulfate has few indoor sources (Sarnat et al. 2002). Table 2 also shows personal-to-out-door ratios (P:O) for PM_2.5_ and SO_4_^2−^, as well as differences between these ratios [P:O (PM_2.5_) − P:O (SO_4_^2−^)]. During winter 1 and winter 2, the difference between P:O (PM_2.5_) and P:O (SO_4_^2−^) was approximately 0.35 (Table 2, next to last row). In contrast, during winter 3, summer 1, and summer 2, this difference was much larger, ranging from 0.75 to 1.55. The larger difference indicates that indoor sources were making a larger contribution to personal PM_2.5_ concentrations during these final three monitoring periods than during the first two monitoring periods. It is unlikely that this was due to recognized (Wallace et al. 2006) indoor sources of PM_2.5_ such as cooking, cleaning, and personal care because these sources should not be significantly stronger during milder weather. Nor is the larger difference due to more time indoors during the final three periods; the seniors were indoors 97% of the time in the winter and 93% of the time in the summer (time in transit included). Instead, the larger difference may be due to greater amounts of indoor ozone–generated SOAs during winter 3, summer 1, and summer 2 than during winter 1 and winter 2 [personal ozone concentrations (Table 2, last row) were 2.5, 5.1, and 4.8 ppb during the former periods and 0.1 and 0.8 ppb during the latter periods].

Additional data in Sarnat et al. (2005) lend further support to this interpretation. Regression results for measurements made during the summer months indicate a slope of 0.35 (0.22–0.47) for personal sulfate regressed on personal ozone, compared with a slope of 0.72 (0.42–1.01) for personal PM_2.5_ regressed personal ozone [see table 3 of Sarnat et al. (2005)]. The much larger slope for the latter pairing is consistent with contributions to personal PM_2.5_ from SOAs generated by ozone-initiated indoor chemistry.

### Health effects of ozone reaction products

Certain ozone reaction products are known to have adverse health effects. For example, formaldehyde has been designated a Group 1 carcinogen in a 2004 International Agency for Research on Cancer evaluation (Cogliano et al. 2005). Acrolein is listed by California as an irritant and carcinogen (California Office of Environmental Health Hazard Assessment 2006). Peroxyactyl nitrate is a known eye irritant (Vyskocil et al. 1998), as are some of the products of ozone/terpene and ozone/isoprene chemistry (Kleno and Wolkoff 2004; Nojgaard et al. 2005). Hydroperoxides formed via the oxidation of terpenes and terpenoids can be potent contact allergens (Gafvert et al. 1994; Karlberg and DoomsGoossens 1997; Matura et al. 2003, 2005; Skold et al. 2002). Leikauf (2002) has listed formaldehyde, acetaldehyde, and acrolein as compounds anticipated to induce or exacerbate asthma. Using a mouse model, Wolkoff and colleagues have demonstrated that ozone/terpene reactions produce strong airway irritants (Clausen et al. 2001; Rohr et al. 2002, 2003a; Wilkins et al. 2001; Wolkoff et al. 1999, 2000). However, an acute exposure study of healthy women exposed for 2 hr to a mixture of volatile organic compounds and ozone (40 ppb) did not result in significant subjective or objective symptoms (Fiedler et al. 2005; Laumbach et al. 2005), suggesting that longer exposures may be necessary to produce a measurable effect.

For some indoor oxidation products, the connection with adverse health effects is more tentative. For example, there is accumulating evidence that outdoor PM_2.5_ adversely affects morbidity and mortality (Dominici et al. 2006; Pope et al. 2002), and SOAs are major constituents of outdoor PM_2.5_. However, SOAs from ozone/terpenoid reactions differ in composition from SOAs generated by outdoor photochemical activity. It is not known how the toxicities of these SOAs compare. An additional consideration is the fact that ozone/terpenoid reactions lead to the co-occurrence of peroxides and submicrometer particles (Docherty et al. 2005; Fan et al. 2005; Li et al. 2002), and this may provide a mechanism to transport some of the peroxides deep into the respiratory tract (Friedlander and Yeh 1998). The consequences of inhaling such oxidation products, some of which are known contact allergens (see above), remain to be evaluated.

Hydroxyl and nitrate radicals, derived from ozone reactions, further react to produce still other oxidation products. Toxic products formed in this manner include malaoxon from the OH oxidation of malathion (Brown et al. 1993) and nitrosoamines and nitro-PAHs from reactions involving nitrate radicals (Gupta et al. 1996; Pitts et al. 1985).

### Average daily indoor intakes of ozone reaction products

Ignoring gas-phase reactions, the ratio of the indoor concentration of ozone oxidation products to the indoor concentration of ozone, [Prod]:[O_3,_*_indr_*], is roughly estimated by


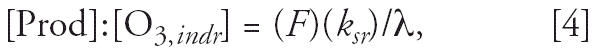


where *k_sr_* and λ are as defined for Equation 1, and *F* is the ratio of the molar emission rate of oxidation products to ozone’s surface removal rate (sometimes called the “formation factor”). Wisthaler et al. (2005) found that for every molecule of ozone removed by surfaces in a simulated aircraft cabin, between 0.2 and 0.25 molecules of oxidized products entered the air. For different types of carpets, Morrison and Nazaroff (2002, figure 5) report that for each ozone molecule removed, 0.1–0.7 aldehyde molecules entered the air. For four different types of surfaces in four different homes, Wang and Morrison (2006) report that for each ozone molecule removed, 0.1–0.4 aldehyde molecules entered the air. In the latter two studies, the total number of oxidized molecules that entered the air is presumably larger than that reported for aldehydes alone because common oxidation products such as formic acid, acetic acid, and acetone were not included in the aldehyde numbers. Although more measurements of *F* are needed in residences and nonresidences, these studies are a beginning. Using a middle estimate of 0.33 for *F* and combining it with a value of 3 hr^−1^ for *k_sr_* and a range of values from 0.5 to 3 hr^−1^ for λ, a conservative estimate for [Prod]:[O_3,_*_indr_*] is 0.33–2. This estimate is termed “conservative” because it considers only airborne products derived from surface chemistry; additional oxidation products derived from gas-phase chemistry (e.g., ozone reacting with terpenes) would result in a larger ratio. Hence, it is reasonable to anticipate that ozone oxidation products are present indoors at concentrations that, on a molar basis, are roughly one-third to twice those of ozone alone. This means that average daily indoor intakes of ozone oxidation products are roughly one-third to twice that of ozone (Figure 1B). Because the products of ozone-initiated chemistry tend to have higher concentrations indoors than outdoors (see above) and that greater time spent indoors overwhelms larger breathing rates outdoors, indoor inhalation intakes of oxidation products tend to be much larger than outdoor intakes of oxidation products.

In a region with moderate outdoor ozone levels, persons doing their own day-to-day house cleaning are estimated to inhale an average of 20 μg/day of formaldehyde and 35 μg/day of SOAs (a large fraction of which are ultrafine particles) as a consequence of ozone-initiated reactions with constituents of cleaning agents and air fresheners [table 5.3 of Nazaroff et al. (2006)]. These values are consistent with intakes of oxidation products estimated in the previous paragraph. Such inhalation intakes add to already existing and often significant intakes of formaldehyde and SOAs from other sources. Furthermore, reactions between ozone and constituents of personal-use products (e.g., perfumes, colognes, hair treatments) emit oxidation products in the vicinity of the breathing zone, resulting in inhalation intakes larger than those predicted if the products were evenly distributed throughout a room (Karamalegos et al. 2005).

## Connections Between Ill Health and Exposure to Indoor Ozone and Its Oxidation Products

### Recent epidemiologic study of mortality in 95 U.S. urban communities

Bell et al. (2004) have used databases from the National Morbidity, Mortality and Air Pollution Study to calculate the average relative rate of mortality associated with short-term ozone concentrations measured at outdoor monitoring stations for 95 U.S. cities between 1987 and 2000. Table 3 presents the 10 cities with the highest percent change in daily mortality per 10-ppb increase in daily ozone and the 10 cities with the lowest percent change. For each of the listed cities, Table 3 also presents the percentage of population growth for the period 1990–2000 and the percentage of residences with central AC. The data were obtained from the U.S. Census Bureau (2006); for selected cities where specific information on central AC was not available, the value is simply listed as being greater than or less than 70% on the basis of comparisons with cities that have similar seasonal dew points and temperatures.

Cities with recent population growth have a larger fraction of new homes and apartments than cities with less growth, and such newer residences tend to have lower air exchange rates (Weisel et al. 2005). Use of central AC is also associated with low air exchange rates (see “Indoor ozone concentrations” above). Conversely, without AC, residents are more likely to open their windows during periods when temperatures are elevated. Hence, compared with older cities that have fewer homes with central AC, newer cities with a higher prevalence of central AC are anticipated to have less outdoor-to-indoor transport and smaller occupant exposures to indoor ozone and the products of ozone-initiated chemistry. Consistent with a connection between such indoor exposures and mortality, 8 of the 10 cities that had the highest percent increase in mortality per 10-ppb increase in ozone had population growth since 1990 < 10% and 8 of the 10 had central AC in < 70% of the structures, whereas 7 of the 10 cities that had the lowest percent increase in mortality per 10-ppb increase in ozone had population growth since 1990 > 10% and 7 of the 10 had central AC in > 70% of its structures (Table 3).

### Other suggestive studies

Levy et al. (2005) conducted an empiric Bayes meta regression to examine the relationship between outdoor ozone concentrations and premature mortality based on 48 estimates from 28 time-series studies. The authors deliberately omitted data from the National Morbidity, Mortality and Air Pollution Study (2006) because other investigators were analyzing these data. In other words, their database was independent of data that are the basis for Table 3. One of the conclusions from their meta regression was that AC prevalence was among the strongest predictors of between-study variability. They go on to state that their results suggest “that the ambient ozone-mortality relationship might be lower in cities with greater prevalence of residential central air conditioning (and therefore lower personal exposure to zone).”

Time-series epidemiologic studies often show seasonal differences in the relative risk from ozone (Ito et al. 2005; Levy et al. 2005; Wong et al. 2001; Zhang et al. 2006). Ozone risk estimates are larger for summer than for winter in New York City; Detroit, Michigan; and Cook County, Illinois (Ito et al. 2005). Conversely, ozone risk estimates are larger for winter than for summer in Houston (Ito et al. 2005), Hong Kong (Wong et al. 2001), and Shanghai (Zhang et al. 2006). In New York, Detroit, and Cook County, there is less outdoor-to-indoor transport during the cold winter, when windows tend to be closed, compared with the warmer summer. Indeed, in Boston, a climatically similar urban area, the association between outdoor ozone and personal ozone has been shown to be weaker in winter than in summer (Sarnat et al. 2005). However, in a southern city such as Houston or a subtropical Asian city such as Hong Kong or Shanghai, there is less outdoor-to-indoor transport during the hot, humid summer, when air conditioners are used extensively and buildings tend to be sealed, compared with the cooler winter when buildings tend to be more open.

## Conclusions

Indoor ozone and products of ozone-initiated indoor chemistry correlate with ozone measured at fixed outdoor sites. I have cited studies indicating that *a*) indoor ozone levels are typically 10–50% of outdoor values, *b*) indoor ozone exposures are typically 45–75% of total exposures, *c*) indoor ozone inhalation intakes are typically 25–60% of total intakes, *d*) indoor sources of chemicals that react with ozone are ubiquitous, *e*) certain oxidation products are known to be toxic and others are anticipated to be toxic, and *f* ) indoor inhalation intakes of these oxidation products are roughly one-third to twice the indoor intakes of ozone and much greater than outdoor intakes of oxidation products. Smaller indoor intakes of ozone are anticipated for people who spend a large fraction of their indoor time in air-conditioned rooms or in rooms with small air exchange rates during periods when outdoor ozone levels are elevated. Smaller indoor intakes of oxidation products are anticipated for people who live in indoor settings with relatively low concentrations of ozone-reactive chemicals, both in the gas phase and associated with surfaces; smaller indoor intakes of oxidation products also result from higher air exchange rates (Equation 4).

By their nature the cited epidemiologic studies include the indoor exposures discussed in this article. Findings from several epidemiologic studies hint at associations between morbidity and mortality and indoor ozone and its oxidation products. However, these studies were not designed to test this hypothesis. Specific studies can be envisioned to evaluate the contribution of indoor ozone and its oxidation products to ill health (Weschler 2004).

Apportioning risk between outdoor and indoor intakes bears on the strategies used to protect public health. Outdoor ozone is harmful to health; outdoor ozone transported indoors is harmful to health; indoor ozone reacts to form products that are also harmful to health, some perhaps more so than ozone. Contrary to popular wisdom, being indoors does not offer clear protection from ozone-related adverse health effects, but it would if ozone were deliberately removed from ventilation air.

Although it has proven difficult and very costly to reduce outdoor ozone concentrations, relatively simple steps can reduce the concentration of indoor ozone and its oxidation products. For example, charcoal filters (Shair 1981; Shields et al. 1999) or chemically impregnated filters (Kelly and Kinkead 1993) could remove a large fraction of ozone in buildings with mechanical ventilation systems. In naturally ventilated buildings, strategies could be employed that reduce ventilation for the portion of the day when ozone is elevated and increase ventilation when ozone levels are lower. The use of products with ozone-reacting constituents could be limited during periods when indoor ozone levels are elevated. Such steps might be especially valuable interventions in schools, hospitals, and childcare centers in regions that continue to experience elevated outdoor ozone concentrations.

## Figures and Tables

**Table 1:** Indoor sources of ozone-reactive chemicals and common stable oxidation products resulting from ozone-initiated reactions with the specified emissions.

Source	Reactive emissions	Major stable products	References
Occupants (exhaled breath, skin oils, personal care products)	Isoprene, nitric oxide, squalene, unsaturated sterols, oleic acid and other unsaturated fatty acids, unsaturated oxidation products	Methacrolein, methyl vinyl ketone, nitrogen dioxide, acetone, 6MHO, geranyl acetone, 4OPA, formaldehyde, nonanal, decanal, 9-oxo-nonanoic acid, azelaic acid, nonanoic acid	Finlayson-Pitts and Pitts (2000), Fruekilde et al. (1998), Thornberry and Abbatt (2004), Taucher et al. (1997), Wisthaler et al. (2005)
Soft woods; wood flooring including cypress, cedar, and silver fir boards; houseplants	Isoprene, limonene, α-pinene, other terpenes and sesquiterpenes	Formaldehyde, 4-AMC, pinonaldehyde, pinic acid, pinonic acid, formic acid, methacrolein, methyl vinyl ketone, SOAs including ultrafine particles	Aoki et al. (2005), Hodgson et al. (2000), Iwashita (2005), Kagi et al. (2005), Atkinson and Arey (2003), SOA references in text
Carpets and carpet backing	4-Phenylcyclohexene, 4-vinylcyclo-hexene, styrene, 2-ethylhexyl acrylate, unsaturated fatty acids and esters	Formaldehyde, acetaldehyde, benzaldehyde, hexanal, nonanal, 2-nonenal	Hodgson et al. (1993), Morrison and Nazaroff (2000, 2002), Weschler et al. (1992a)
Linoleum and paints/polishes containing linseed oil	Linoleic acid, linolenic acid	Propanal, hexanal, nonanal, 2-heptenal, 2-nonenal, 2-decenal, 1-pentene-3-one, propionic acid, *n*-butyric acid	Andersson et al. (1996), Clausen et al. (2005), Wolkoff (1995)
Latex paint	Residual monomers	Formaldehyde	Reiss et al. (1995a)
Certain cleaning products, polishes, waxes, air fresheners	Limonene, α-pinene, terpinolene, α-terpinene and other terpenes, α-terpineol, linalool, linalyl acetate and other terpenoids, longi-folene and other sesquiterpenes	Formaldehyde, acetaldehyde, glycoaldehyde, formic acid, acetic acid, hydrogen and organic peroxides, acetone, benzaldehyde, 4-hydroxy-4-methyl-5- hexen-1-al, 5-ethenyl-dihydro-5-methyl-2(3*H*)-furanone, 4-AMC, SOAs including ultrafine particles	Aoki et al. (2005), Destaillats et al. (2006a), Englund et al. (1996), Liu et al. (2004), Nazaroff and Weschler (2004), Shu et al. (1997), Singer et al. (2006), Wolkoff et al. (1998), SOA references in text
Natural rubber adhesive	Isoprene, terpenes	Formaldehdye, methacrolein, methyl vinyl ketone	Aoki et al. (2005), Atkinson and Arey (2003)
Photocopier toner, printed paper, styrene polymers	Styrene	Formaldehyde, benzaldehyde	Aoki et al. (2005), Wolkoff et al. (1993), Wolkoff (1999)
Environmental tobacco smoke	Styrene, acrolein, nicotine	Formaldehyde, benzaldehyde, hexanal, glyoxal, *N*-methylformamide, nicotinaldehyde, cotinine	Destaillats et al. (2006b), Shaughnessy et al. (2001)
Soiled clothing, fabrics, bedding	Squalene, unsaturated sterols, oleic acid and other unsaturated fatty acids	Acetone, geranyl acetone, 6MHO, 4OPA, formaldehyde, nonanal, decanal, 9-oxo-nonanoic acid, azelaic acid, nonanoic acid	Fruekilde et al. (1998), Thornberry and Abbatt (2004), Wisthaler et al. (2005)
Soiled particle filters	Unsaturated fatty acids from plant waxes, leaf litter, and other vegetative debris; soot; diesel particles	Formaldehyde, nonanal, and other aldehydes; azelaic acid; nonanoic acid; 9-oxo-nonanoic acid and other oxo-acids; compounds with mixed functional groups (== O, –OH, and –COOH)	Bekö et al. (2006), Hyttinen et al. (2003, 2006), Thornberry and Abbatt (2004)
Ventilation ducts and duct liners	Unsaturated fatty acids and esters, unsaturated oils, neoprene	C_5_ to C_10_ aldehydes	Morrison et al. (1998)
“Urban grime”	Polycyclic aromatic hydrocarbons	Oxidized polycyclic aromatic hydrocarbons	Kahan et al. (2006)
Perfumes, colognes, essential oils (e.g. lavender, eucalyptus, tea tree)	Limonene, α-pinene, linalool, linalyl acetate, terpinene-4-ol, γ-terpinene	Formaldehyde, 4-AMC, acetone, 4-hydroxy-4-methyl-5-hexen-1-al, 5-ethenyl-dihydro-5-methyl-2(3*H*) furanone, SOAs including ultrafine particles	Chao et al. (2005), Karamalegos et al. (2005), Shu et al. (1997), SOA references in text
Overall home emissions	Limonene, α-pinene, styrene	Formaldehyde, 4-AMC, pinonaldehdye, acetone, pinic acid, pinonic acid, formic acid, benzaldehyde, SOAs including ultrafine particles	Hodgson et al. (2000), Park and Ikeda (2006), Atkinson and Arey (2003), SOA references in text

Abbreviations: 4-AMC, 4-acetyl-1-methyl-cyclohexene; 6MHO, 6-methyl-5-heptene-2-one; 4OPA, 4-oxopentanal.

**Table 2:** Personal and outdoor concentrations (μg/m^3^) of PM_2.5_ and sulfate for senior citizens in Boston, as well as P:O ratios for each, the difference between these [P:O (PM_2.5_) − P:O (SO_4_^2−^)], and the corresponding personal ozone concentrations (pbb).^a^

	Winter 1 (5 subjects)	Winter 2 (4 subjects)	Winter 3 (5 subjects)	Summer 1 (9 subjects)	Summer 2 (5 subjects)
Personal PM_2.5_	10.8 (*n* = 40)	15.4 (*n* = 41)	16.2 (*n* = 51)	17.8 (*n* = 106)	20.5 (*n* = 59)
Outdoor PM_2.5_	13.1 (*n* = 13)	15.5 (*n* = 12)	6.5 (*n* = 15)	11.9 (*n* = 11)	13.3 (*n* = 13)
P:O (PM_2.5_)	0.82	0.99	2.49	1.50	1.54
Personal SO_4_^2−^)	1.6 (*n* = 51)	2.6 (*n* = 42)	1.6 (*n* = 56)	2.7 (*n* = 104)	3.3 (*n* = 59)
Outdoor SO_4_^2−^)	3.4 (*n* = 13)	4.2 (*n* = 12)	1.7 (*n* = 13)	3.6 (*n* = 8)	4.2 (*n* = 12)
P:O (SO_4_^2−^)	0.47	0.62	0.94	0.75	0.79
P:O (PM_2.5_) − P:O (SO_4_^2−^)	0.35	0.37	1.55	0.75	0.75
Personal O_3_	0.1 (*n* = 50)	0.8 (*n* = 43)	2.5 (*n* = 57)	5.1 (*n* = 106)	4.8 (*n* = 50)

Data from Sarnat et al. (2000, table 2).

a PM_2.5_, SO_4_^2−^, and O_3_ concentrations are based on 24-hr samples. *n* = total number of measurements for each condition.

**Table 3:** Cities with highest and lowest percent change in daily mortality per 10-ppb increase in daily ozone^a^, percentage of population growth^b^, and percentage of housing units with central AC^b^.

	Change in daily mortality (%)	Population growth, 1990–2000 (%)	Central AC (%)
Ten cities with highest percent change (of 95 cities)			
New York City	1.7	9.4	16
Newark, NY	1.3	−0.6	47
Philadelphia, PA	1.3	−4.3	50
Cincinnati, OH	1.2	−9.0	66
Dallas/Ft. Worth, TX	1.1	18.5	91
Shreveport, LA	1.0	0.8	> 70
Chicago, IL	0.9	4.0	62
Syracuse, NY	0.9	−10.1	< 70
Colorado Springs, CO	0.9	28.4	< 70
Worcester, MA	0.9	1.7	< 70
Average	1.1	3.9	—
Ten cities with lowest percent change (of 95 cities)			
Orlando, FL	−0.2	12.9	> 70
Denver, CO	0.0	18.6	50
San Antonio, TX	0.1	22.3	78
Las Vegas, NV	0.1	85.2	> 70
Little Rock, AR	0.1	4.2	> 70
Lexington, KY	0.2	15.6	> 70
Birmingham, AL	0.2	−8.7	77
San Diego, CA	0.2	10.2	34
St. Petersburg, FL	0.2	4.0	87
Lafayette, IN	0.3	28.9	< 70
Average	0.1	20.0	—

− negative value.

a Data from Bell et al. (2004).

b Data from U.S. Census Bureau (2006).
